# Patients’ expectations of and experiences with psychosocial care needs in perioperative nursing: a descriptive study

**DOI:** 10.1186/s12912-023-01451-1

**Published:** 2023-09-05

**Authors:** Kari Ingstad, Mona K. Pedersen, Lisbeth Uhrenfeldt, Preben U. Pedersen

**Affiliations:** 1 Faculty of Nursing and Health Science, Nord University, Pb. 93, Levanger, 7601 Norway; 2 Centre for Clinical Research, North Denmark Regional Hospital, Hjoerring, Denmark; 3 Department for Clinical Medicine, Aalborg University, Aalborg, Denmark; 4 Nord University Faculty of Nursing and Health Science, Nord University, Bodø, Norway; 5 Institute of Regional Health Research, Southern Danish University, Ortopedic dep., Lillebaelt University Hospital, Kolding, Denmark; 6 Centre of Clinical Guidelines, Department of Clinical Medicine, University of Aalborg, Aalborg, Denmark

**Keywords:** FoC, Inpatients, Missed Care, Patients, Perioperative care, Psychosocial Care, Person-centered, Subjective importance, Perceived reality, Healthcare personnel

## Abstract

**Background:**

Meeting inpatients’ psychosocial care needs is essential for their wellbeing, recovery, and positive experiences. This study aimed to describe and compare surgical inpatients’ subjective perceptions of the importance of fundamental psychosocial and overall care received.

**Methods:**

A descriptive study with a convenient sample was conducted from September 2019 to April 2020. A total of 194 surgical inpatients from Norway and Denmark answered a perioperative user participation questionnaire on the day of discharge. The questionnaire was previously face- and content validated. The questionnaire assessed patients’ sociodemographic characteristics and four dimensions of fundamental care domains: Psychosocial, Relational, Physical, and System level. This study reports the results from the psychosocial domain. Descriptive statistics including frequencies, percentages, means, and standard deviations were used to analyze background information variables. The congruency between participants’ expectations of and experiences with psychosocial care is presented.

**Results:**

The inpatients expected (and experienced) the healthcare personnel to treat them with respect and dignity, and to be involved and informed throughout their perioperative care. The average ratings regarding these aspects of psychosocial care needs were 72.1–93.8%. There was congruency between patients’ perceptions of the subjective importance (SI) of psychosocial fundamental care and their perceived reality (PR) of care. Congruency between high SI and high PR ranged from 59.1 to 92.2%, and congruency between low SI and low PR ranged from 0 to 6.6%. Incongruency between SI and PR varied between 5.9 and 39.6% and was mainly related to higher PR than SI. We found no association between education level, sex, length of stay, age, and patient expectations of or experiences with psychosocial care needs.

**Conclusions:**

Surgical inpatients in Norway and Denmark experience respectful and dignified treatment, and they feel involved and informed in their perioperative care. It is important to include patient perspectives in further research to avoid missed care and disconnection between what patients prefer and what healthcare personnel plan to do. Understanding patient preferences might also lead to less stress and workload for healthcare personnel.

## Background

Meeting the psychosocial care needs of patients is essential for ensuring their optimal safety, recovery, and positive experiences [1]. Interventions improving psychosocial care, such as clinician communication training and on-site visits for context analysis and problem-solving, have been demonstrated to be effective to reduce suffering [2] and improve quality of life [3] in patients with dementia or cancer. Furthermore, the effectiveness of psychosocial techniques to decrease post-operative pain and improve perioperative clinical care in orthopedic surgery has been reported in a meta-analysis [4]. The results indicated that psychosocial interventions may reduce perioperative side effects and improve recovery. Patients who benefit from psychosocial care are believed to make less use of other healthcare services and be more compliant with treatments or lifestyle recommendations, resulting in better overall health [5, 6].

Inpatients experience a wide range of psychosocial healthcare needs. In this study, we have explored the psychosocial care needs within the Fundamentals of Care (FoC) framework. The FoC framework outlines what is involved in the delivery of safe, effective, high-quality fundamental care, and what this care should look like in any healthcare setting and for any care recipient. The FoC framework comprises three interrelated dimensions: relationship, integrated fundamental care, and context. The framework is usually illustrated by three concentric circles that integrate the core relational elements at the center with the system requirements related to the delivery of care in the outer circle, also called the context of care. Unique to the framework is the second/middle circle, which focuses on assessing and meeting a patient’s essential physical and psychosocial needs to ensure that their care needs are met. The crucial point of the framework is the relationship between the nurse and the patient concerning fundamental care delivery so that the physical and psychosocial dimensions of the fundamentals of care are mediated in each interaction between the nurse and the patient [7].

According to the FoC framework, the recipients’ psychosocial care needs are communication, being involved and informed, respect, education and information, dignity, emotional wellbeing, having values and beliefs considered and respected, and privacy [1, 7]. Two scoping reviews concluded that communication is a key aspect of unmet needs identified by many patients across the studies [8, 9]. Furthermore, patients and relatives miss emotional support [9–12], and patients want more information about their care plans and the procedures they are going to undergo and discussion with nurses about their medication and the organization and internal rules of the ward [8]. Unmet needs are related to missed care—defined as any aspect of patient care that is omitted or delayed—and are receiving increasing attention [9]. Respect for privacy is an area of missed care identified by patients [8]. Privacy of information was reported as an unmet care need by many patients who felt that their confidentiality was not always respected during their conversations with nurses [8, 13].

The focus on efficacy and productivity in hospitals increases pressure on nurses’ care [14–16]. Studies have shown that nurses in hospital wards are mainly focused on physical care and do not take the time to care for patients’ psychosocial and relational needs [17]. The high-tempo culture of a surgical ward challenges patients’ safety and leads to patients not receiving optimal physical or emotional support [18].

Missed care could be considered from different perspectives; for example, representing both patients’ and nurses’ evidence as background for decision-making and actions. It is important to consider patients’ perspectives throughout the healthcare process because patients are the principal end users of the services that health organizations provide [8]. However, research about missed care from the patients’ perspective is very limited [8, 9]. Investigating missed nursing care only from the perspectives of the professionals, without considering the person as a whole, can result in technical activities of nursing care being poorly targeted to the real need of the patient [8]. Further, patients and nurses may have different priorities [19]. In a prior study, nurses perceived that they delivered care focusing on autonomy, informed consent, and privacy more often than what patients perceived [20].

This study contributes to the existing literature by taking a person-centered perspective [21] and measures both patients’ expectations of and experiences with psychosocial care and their relationship. Patients’ expectations have been described as the subjective importance (SI) of aspects of care, while patients’ experience of the care received has been described as patients’ perceived reality (PR). Combining measurements for SI and PR allows to identify congruence between SI and PR and identify gaps that can be addressed and researched [22]. The Perioperative User Participation (POUP) questionnaire has been developed and validated to measure four domains within the FoC framework. In this study, results from the psychosocial domain are reported; thus, the findings contribute to knowledge concerning surgical inpatients’ expectations of and experiences with psychosocial care.

### Aim

This study aimed to establish a baseline description of Norwegian and Danish surgical inpatients’ perceptions of the SI and PR of psychosocial fundamental care, as a part of a study involving users of health care to improve the offered quality of care.

## Methods

This descriptive study was designed to explore both Norwegian and Danish adult patients’ experiences of psychosocial care during hospitalization for surgical treatment. Psychosocial care was operationalized within the FoC framework [7, 23].

### Sampling and study population

A convenient sample of 194 elective hospitalized patients answered the POUP questionnaire [22]: 117 from Denmark and 77 from Norway. Data were collected from one hospital in Norway (four units) and one hospital in Denmark (three units), from September 2019 to April 2020. Both hospitals were publicly funded. The inclusion criteria were as follows: (i) adults aged ≥ 18 years, (ii) admitted for an elective surgical procedure, (iii) able to speak and understand Norwegian and/or Danish, and (iv) volunteering. The exclusion criteria were patients unable to provide written informed consent.

The patients were asked to respond to the POUP questionnaire on the day of hospital discharge. The questionnaires were distributed in a closed envelope by a nursing student (Norway) or by either a ward nurse or a research assistant (Denmark) and returned in a closed envelope just before discharge. The sealed envelopes containing the POUP questionnaires were initially stored in the leader’s office and the collected data were subsequently stored in a locked cupboard. Afterwards, only the four authors and one research assistant had access to the collected data.

### Instruments

The POUP questionnaire, based on a theoretical person-centered model of quality of care—the Quality of Care from Patients’ Perspective (QPP [24])—was constructed to measure what elective adult surgical patients value as important and how they assess the care they have received. The theoretical foundation of the FoC framework was used to guide the team in the questions that should be developed or content that should be added to the QPP questionnaire, based on our specific research aims.

The POUP questionnaire was designed to assess four psychometric scales, one for each of the FoC domains: Psychosocial (three FoC-subdomains and 15 items: being involved (three items), respected/dignified (five items), and informed (seven items)), Relational (five FoC-subdomains and 13 items), Physical (seven FoC-subdomains and 24 items) and System level (three FoC-subdomains and ten items). In total, the questionnaire consisted of 62 items and one open-ended question allowing participants to comment on any aspect of the admission and care [22]. In addition, the questionnaire consisted of eight background questions including age, sex, highest education level, marital status, employment status, waiting time for admission, and length of stay (LOS). Surgical ward patients were admitted to undergo orthopedic, abdominal, urological, or gynecological surgery.

The entire scale and each item have been validated. It has been face- and content-validated with patients and clinical nurse experts [24]. A group of 68 patients in the post-operative wards in Norway and Denmark validated the relevance of the questions in the four scales to be between 78 and 92%. The internal consistency was assessed to be between α = 0.78 and α = 0.84 for the combined PR items from Denmark and Norway. For SI measures, the combined internal consistency (α) ranged from 0.58 to 0.92 [22]. No differences in scores between countries were detected.

SI and PR were both measured on ordinal scales from 0 to 4. SI scales asked patients to assess items that started with, ‘This is how important this is to me…’ (e.g., To be able to have a conversation in privacy with a nurse; 0 = *not relevant* to 4 = *very important for me*. For PR, patients were asked, ‘This is what I experience…’ (e.g., To be able to have a conversation in privacy with a nurse; 0 = *not relevant* to 4 = *fully agree* [22]. The scores ranged from 0 to 60. The reliability of POUP was assess calculating the internal consistency. For the psychosocial scale yielded good to moderate reliability with α = 0.81 (CI95%: 0.70–0.89) for PR and 0.64 (CI95%: 0.33-0.84) for SI. In the present population, the Internal consistency calculation α = 0.78 for PR and 0.89 for SI, indicating acceptable internal consistency and good reliability.

### Statistical analyses

Data were analyzed using SPSS, Version 26. Descriptive statistics including frequencies, percentages, means, and standard deviations (SDs) were used to analyze background information variables. The ratings for each item were calculated summarizing scores from each item on the scale. The difference between SI and PR scores was tested using independent sample t-tests or one-way analyses of variance. A 95% confidence interval (CI) is given. Statistical significance was considered at p < .05.

Ordinal scale data are presented as numbers and frequencies. Patients’ scoring of the relevance of SI and PR items within a psychometric scale is presented as the mean frequency and minimum and maximum frequencies of items scored as ‘not relevant’ within the psychometric scales. By measuring PR and SI, a new variable can be generated as the congruence between patients’ perceptions of the care they received (the PR) and their perceptions of how important the various care aspects are to them (the SI of the care aspects). Table 1 illustrates how the scores have been recoded and how the new variable can be interpreted. Incongruency is present when one of the scales had a high score and the other a low score.

**Table 1:** Calculation and interpretation of congruency score

	Perceived Reality High PR scores (4 or 3)	Perceived Reality Low PR scores (2 or 1)
**Subjective** **Importance** **High** **SI scores (4 or 3)**	HIGH-HIGH: Patients report that there is congruency between the care they receive and their assessments of the importance of the aspect of care.	HIGH-LOW: Patients report that there is incongruency between the care they receive and their assessments of the importance of the aspect of care.
**Subjective Importance** **Low SI** **SI scores (2 or 1)**	LOW-HIGH: Patients report that there is incongruency between the care they receive and their assessments of the importance of the aspect of care.	LOW-LOW: Patients report that there is congruency between the care they receive and their assessments of the importance of the aspect of care.

### Ethical considerations

The perioperative care research study, which this study is part of, was registered with the Norwegian Centre for Research Data (no. 61,358). A computing agreement was signed between Nord University (Head of faculty) and the participating institutions (Leader of Danish Centre for Clinical Guidelines, Aalborg University, and Centre for Clinical Research, North Denmark Regional Hospital).

Oral and written information about the objectives and the procedures of the study was given to each participant by a nursing student (Norway) or by either a ward nurse or a research assistant (Denmark) on the day of discharge. The same professionals collected the participants’ written informed consent forms, which were stored separately in locked cupboards in the leader’s office. The questionnaires were returned in a blank, anonymous envelope at discharge. The patients were assured that participation in this study was voluntary and anonymous and that declining to participate would not affect their care and treatment and that all data were held confidential and accessed only by members of the research team. Furthermore, patients were informed that if they changed their mind, they could return a blank questionnaire. Finally, all participants provided written informed consent.

## Results

### Respondent characteristics

The study population is described in Table 2. Overall, no significant differences between the countries were found regarding sex (p < .05) and age (p < .001). Most respondents in both countries were women. The mean age was 55.8 years, ranging from 18 to 97 years.

**Table 2:** Demographic description of patients included in the baseline test in Norway, Denmark, and in total

	Norway	Denmark	Total
	n = 77	n = 117	N = 194
**Sex**			
Male (%)	25 (32.5)	44 (37.6)	69 (35.6)
Female (%)	52 (67.5)	73 (62.4)	125 (64.4)
**Age (years)**			
Mean (SD)	59.6 (15.7)	53.2 (17.4)	55.8 (17.0)
Min–max	(18–89)	(24–97)	(18–97)
**Marital status**			
Living alone (%)	16 (20.8)	19 (16.2)	35 (18.0)
**Education**			
Basic	10 (13.0)	53 (45.3)	63 (32.5)
High school	31 (40.3)	38 (32.5)	69 (35.6)
University	26 (33.8)	24 (20.5)	50 (25.8)
Not reported	10 (13.0)	2 (1.7)	12 (6.2)
**Civil status**			
Studying	2 (2.6%)	2 (1.7)	4 (2.1)
Working/unemployed	26 (33.8)	56 (47.9)	82 (42.3)
Retired/sick pension	33 (42.9)	42 (35.9)	75 (38.7)
Other	11 (14.3)	17 (25.5)	28 (14.4)
Not reported	5 (6.5)	0	5 (2.6)
**Waiting for admission**			
< 7 days	12 (15.6%)	29 (24.8)	41 (21.1)
7–30 days	16 (20.8)	18 (15.4)	34 (17.5)
31–90 days	26 (33.8)	29 (24.8)	55 (28.4)
91–180 days	16 (20.8)	29 (24.8)	45 (23.2)
> 180 days	2 (2.6)	8 (6.8)	10 (5.2)
Not reported	5 (6.5)	4 (3.4)	9 (4.6)
**Length of stay**			
1 day	21 (27.3)	78 (66.7)	99 (51.0)
2–3 days	36 (46.8)	18 (15.4)	54 (27.8)
4–9 days	13 (16.9)	20 (17.1)	33 (17.0)
> 9 days	4 (5.2)	0	4 (2.1)
Not reported	3 (3.9)	1 (0.9)	4 (2.1)
**Surgical procedure**			
Abdominal	19 (24.7)	73 (62.4)	92 (47.4)
Gynecology (including Caesarean section)	27 (35.1)	43 (36.8)	70 (36.1)
Orthopedic	15 (19.5)	0	15 (7.7)
Urology	13 (16.9)	0	13 (6.7)
Not reported	3 (3.9)	1 (0.9)	4 (2.1)

There were significant differences between the countries according to civil status, education level, working situation, and LOS in hospital. More respondents from Norway were living alone (p = .012), respondents from Norway had more education (p < .001), and more Norwegian (vs. Danish) participants were retired (p = .04). The average LOS in hospital was higher among respondents from Norway than those from Denmark (p < .001). More than half the participants stayed at the hospital 1 day (n = 99, 51%), while one-third (n = 54, 33%), stayed 2 days or longer. Four Norwegian participants had a LOS exceeding 9 days.

### Patients’ experiences around psychosocial care needs

This study investigated inpatients’ experiences and preferences regarding psychosocial care during the perioperative period. Psychosocial care was operationalized into subdomains such as Respect/Dignity, Involved, and Informed. The mean scores for SI-psychosocial and PR-psychosocial scales were SI = 43.8 (SD = 12.6; 95% CI: 42.0–45.6) and 41.3 (SD = 7.7, 95% CI: 40.2–42.4), respectively (p < .001).

No significant relationships were found between the total SI and PR score for the following independent sociodemographic variables: country, sex, civil status, education, surgical procedure, and LOS.

The frequencies and SI and PR ratings for single items are presented according to the subdomains in Tables 3, 4 and 5.

**Table 3:** Surgical inpatients’ congruency between expectation and experiences of respect/dignity

Question	Subjective importance			Perceived reality			Congruency between them	
	Important n (%) (95% CI)	Not important n (%) (95% CI)	Not relevant/missing n (%) (95% CI)	Agree n (%) (95% CI)	Do not agree n (%) (95% CI)	Not relevant/missing n (%) (95% CI)	Congruency High (H) Low (L) n (%) (95% CI)	Incongruency n (%) (95% CI)
I found that my needs in the operating room were considered (e.g., clothing, pain relief, placement)	155 (79.9) (74–85)	17 (8.8) (5–14)	22 (11.3) (7–17)	170 (87.7) (82–92)	8 (4.1) (2–8)	16 (8.3) (5–13)	H: 152 (88.4) (83–93) L: 4 (2.3) (1–6)	16 (9.3) (5–15)
My private boundaries were respected during my stay in the ward	112 (57.7) (50–65)	30 (15.5) (11–21)	52 (26.8) (21–34)	144 (74.2) (67–80)	12 (6.2) (3–11)	38 (19.6) (14–26)	H: 99 (72.8) (65–80) L: 2 (1.5) (0–5)	35 (25.7) (19–34)
My private boundaries were respected in the operating room	115 (59.3) (52–66)	31 (16.0) (11–22)	48 (24.7) (19–31)	146 (75.2) (69–81)	9 (4.7) (2–9)	39 (20.1) (15–26)	H: 78 (67.2) (58–76) L: 0 (0.0) (0–3)	38 (32.8) (24–42)
My private boundaries were respected in the recovery room	111 (57.2) (50–64)	31 (16.0) (11–22)	52 (26.8) (21–34)	140 (72.1) (65–78)	8 (4.2) (2–8)	46 (23.7) (18–33)	H: 99 (73.3) (65–81) L: 0 (0.0) (0–3)	36 (26.7) (19–35)
My privacy was respected during hospitalization	155 (79.9) (74–85)	14 (7.2) (4–12)	25 (12.9) (9–18)	169 (87.1) (82–91)	6 (3.1) (1–7)	19 (9.9) (6–15)	H: 151 (89.9) (84–94) Low: 2 (1.2) (0–4)	15 (8.9) (5–14)

**Table 4:** Surgical inpatients’ congruency between expectation and experiences of involvement

Question	Subjective importance			Perceived reality			Congruency between them	
	Important n (%) (95% CI)	Not important n (%) (95% CI)	Not relevant/missing n (%) (95% CI)	Agree n (%) (95% CI)	Do not agree n (%) (95% CI)	Not relevant/missing n (%) (95% CI)	Congruency High (H) Low (L) n (%) (95% CI)	Incongruency n (%) (95% CI)
Before discharge, I was instructed about when and how to deal with suture removal, skin care, training, etc. when I got home	161 (83.0) (77–88)	7 (3.6) (2–8)	26 (13.4) (9–19)	161 (83.0) (77–88)	10 (5.2) (3–10)	23 (11.9) (8–17)	H: 153 (92.2) (87–96) L: 3 (1.8) (0–5)	10 (6.0) (3–11)
I had good opportunities to participate in decisions that concerned my care and treatment	136 (70.1) (63–76)	21 (10.9) (7–16)	37 (19.0) (14–25)	147 (75.7) (69–82)	18 (9.2) (6–14)	29 (14.9) (10–21)	H: 128 (82.1) (75–88) L: 7 (4.5) (2–9)	21 (13.4) (9–20)
I gained knowledge of what to expect during the course before I was admitted to the hospital	45 (23.2) (17–30)	107 (55.1) (48–62)	42 (21.6) (16–28)	141 (72.7) (66–79)	21 (10.8) (7–16)	32 (16.5) (12–22)	H: 88 (59.1) (51–67) L: 2 (1.3) (0–5)	59 (39.6) (32–48)

**Table 5:** Surgical inpatients’ congruency between expectation and experiences of information

Question	Subjective importance			Perceived reality			Congruency between them	
	Important n (%) (95% CI)	Not important n (%) (95% CI)	Not relevant/missing n (%) (95% CI)	Agree n (%) (95% CI)	Do not agree n (%) (95% CI)	Not relevant/missing n (%) (95% CI)	Congruency High (H) Low (L) n (%) (95% CI)	Incongruency n (%) (95% CI)
Before the operation, I received good information about the risk associated with the procedure	160 (82.5) (76–88)	24 (12.4) (8–18)	10 (5.2) (3–9)	163 (84.0) (78–89)	25 (12.9) (9–18)	6 (3.1) (1–7)	H: 147 (80.3) (74–86) L: 12 (6.6) (3–11)	24 (13.1) (9–19)
In the operating room, I was given good information about what would happen	166 (85.6) (80–90)	19 (9.8) (6–15)	9 (4.6) (6–15)	182 (93.8) (89–97)	10 (5.1) (3–9)	2 (1.0) (0–4)	H: 165 (89.2) (84–93) L: 9 (4.9) (5;2–9)	11 (5.9) (3–10)
Before/after the operation, I received good information about the expected result of my operation and care	174 (89.7) (85–94)	8 (4.1) (2–8)	12 (6.2) (3–11)	171 (88.2) (83–92)	15 (7.8) (4–12)	8 (4.1) (2–8)	H:163 (90.0) (85–94) L:5 (2.8) (3;1–6)	13 (7.2) (4–12)
I was given good information about why the operation should be performed	163 (84.0) (78–89)	14 (7.2) (4–12)	17 (8.7) (5–14)	174 (89.7) (85–94)	10 (5.1) (3–9)	10 (5.1) (3–9)	H: 158 (89.3) (89;84–93) L: 5 (2.8) (3;1–6)	14 (7.9) (4–13)
I was given good information about what I could do myself to get well through the surgery	159 (81.9) (76–87)	16 (8.2) (5–13)	19 (9.8) (6–15)	164 (84.6) (79–89)	16 (8.2) (5–13)	14 (7.3) (4–12)	H: 150 (86.7) (81–91) L: 8 (4.6) (2–9)	15 (8.7) (5–14)
I was given good information about medical treatment so that I understood how it worked and how it should be taken	157 (80.9) (75–86)	10 (5.2) (3–9)	27 (14.0) (9–20)	164 (84.5) (79–89)	9 (4.6) (2–9)	21 (10.8) (7–16)	H: 147 (89.6) (84–94) L: 1 (0.6) (0–3)	16 (9.8) (6–15)
I received good information about everyday life after surgery	165 (85.1) (79–90)	17 (8.7) (5–14)	12 (6.1) (3–11)	164 (84.5) (79–89)	25 (12.9) (9–18)	5 (2.5) (1–6)	H: 150 (82.9) (77–88) L: 8 (4.4) (2–9)	23 (12.7) (8–18)

### Surgical inpatient perceptions of respect/dignity

This domain considered five questions regarding how inpatients’ needs in the operating room were considered (e.g. clothing, pain relief, placement; Q17), how private boundaries were respected during the stay in the ward (Q55), operating room (Q56), recovery room (Q57), and during hospitalization (Q58).

As shown in Table 3, between 57.2% and 79.9% of participants perceived that these care aspects were important, and between 72.1% and 87.7% perceived that they were respected/dignified. Similarly, 87.7% of participants perceived that their needs in the operating room were considered, and 72.1% perceived that their boundaries were respected in the recovery room.

Between 3.1% and 6.2% of the respondents experienced that they were not respected/dignified.

For SI, between 11.3% and 26.8% of participants perceived the question as not relevant (missing), and between 8.3 and 23.7 of patients perceived the PR questions as not relevant (missing). We found congruency between patients’ SI and PR scores: 90.7 (Q17), 74.3 (Q55), 67.2 (Q56), 73.3 (Q57), and 91.1 (Q58). Incongruency was related to higher PR than SI.

### Surgical inpatients’ perceptions of involvement

This domain considered three questions regarding information on how inpatients were instructed about when and how to deal with aspects such as suture removal, skin care, and training once discharged (Q15); their opportunities to participate in decisions that concerned their care and treatment (Q16); and if they wished they knew more about what to expect before they were admitted to the hospital (Q59).

As shown in Table 4, between 23.2% and 83.0% of participants perceived that these care aspects were important, and between 72.7% and 83.0% perceived that they were involved; 72.2% perceived that they gained knowledge before they were admitted to the hospital on what to expect during the course and 83.0% perceived that they were instructed before discharge about when and how to deal with aspects such as suture removal, skin care, and training once back home.

According to the SI scale, between 13.4% and 21.6% scored these questions as not relevant (or missing), and between 11.9% and 16.5% of patients perceived the PR questions as not relevant (or missing).

We found congruency between SI and PR scores: 94.0 (Q15), 86.6 (Q16), and 60.4 (Q59). Incongruency was related to higher PR than SI.

### Surgical inpatients’ perceptions of being informed

This domain considered seven questions regarding how inpatients received information about the risk associated with the procedure (Q8), what would happen in the operating room (Q9), the expected results of the operation (Q10), why the operation should be performed (Q11), what they could do to get well through the surgery (Q12), medical treatment (Q13), and everyday life after surgery (Q14).

As shown in Table 5, between 80.9% and 89.7% of participants perceived that these care aspects were important, and between 84.0% and 93.8% perceived that they were informed; 93.8% perceived that they were given good information in the operating room, and 84.0% perceived that they received good information about the risk associated with the procedure before the operation.

According to the SI scale, between 4.6% and 14.0% scored these questions as not relevant (or missing), and between 1.0% and 10.8% of patients perceived the PR questions as not relevant (or missing).

We found congruency between SI and PR scores: 86.9 (Q8), 94.1 (Q9), 92.8 (Q10), 92.1 (Q11), 91.3 (Q12), 90.2 (Q13), and 87.3 (Q14). Incongruency (> 10%) was related to higher PR than SI.

## Discussion

This study explored Norwegian and Danish adult patients’ expectations of and experiences with psychosocial perioperative care during hospital admission for surgical treatment. The patients generally reported that they were treated with respect and dignity. They were also involved and informed about their perioperative care. The average congruency ratings regarding these aspects of psychosocial care needs were 72.1–93.8%. The data in our study were collected on the day of hospital discharge. Patients’ expectations and experiences may change when they return home. Interviews with patients after discharge may give further information and provide diverse results.

Patients’ satisfaction with their psychosocial perioperative care was partially confirmed in previous national surveys. A national survey in Norway showed that patients provide relatively good feedback about their experiences from hospital stays; however, the results also indicate that patients will benefit from improvements in hospitals’ practice regarding discharge and collaboration with municipal services [25]. In Denmark, 86% of surveyed patients with physical illnesses were satisfied with the treatment they received at the hospital [26]. The highest score in the Danish survey was related to friendly and accommodating staff and information received [26].

The POUP instrument provides unique insight into congruency and incongruency between SI and PR of care from patients’ perspectives. As shown in Table 1, congruency may manifest in two situations: HIGH-HIGH, patients report that there is congruency between the care they receive and their assessments of the importance of the aspect of care; and LOW-LOW, patients report that there is congruency between the care they receive and their assessments of the importance of the aspect of care. In the latter situation, patients assess the aspect of care as not important and report that they have received little or no care.

However, congruence only illustrates that care is aligned with patients’ expectations and/or healthcare providers’ knowledge assessment of the importance of the specific care aspect. This might not necessarily be aligned with the evidence for the specific care aspect. This could be used to highlight potential areas of improvement within evidence-based practice, as healthcare personnel have spent resources on delivering care which is not based on evidence but solely on patients’ perceptions, which may be biased. Information or education might change future patients’ SI on a specific care aspect. However, if the decision from healthcare personnel is based on biased or inaccurate evidence on the care aspect, then healthcare personnel would miss the opportunity to deliver care and inform or educate patients about why the care aspect is important for them.

Incongruency may manifest in two scenarios. First, HIGH-LOW, when patients report that there is incongruency between the care they receive and their assessments of the importance of the aspect of care; patients assess the aspect of care as important and the PR as low. Second, LOW-HIGH, when patients assess the aspect of care as not important but do report that they have received care. The latter scenario may be indicative of an unnecessary use of resources, by the healthcare personnel, on the care aspects.

Our study showed ample congruency between SI and PR regarding psychosocial needs. Congruency between high SI and high PR ranged from 59.1 to 92.2%, whereas congruency between low SI and low PR ranged from 0 to 6.6%. Incongruency between SI and PR varied between 5.9% and 39.6% and was mainly related to higher PR than SI. Moreover, 13 of the 15 items showed PR scores higher than the respective SI scores.

An eventual discrepancy between patients’ and healthcare personnel’s experiences, when PR is higher than SI, may be related to overly high professional standards or healthcare personnel spending time/focusing on less important aspects for patients. Nurses’ professional standards can lead them to do more than patients need and expect. Other studies show that nurses experience personal ethical responsibility [27] and responsibility for continuity in perioperative practice [28]. Ethical values and standards above patients’ expectations may lead to unnecessary workload and exhaustion. For future patients, nurses might consider changing practice and informing or educating patients about why the care aspect is either unimportant or important for the patient in the present situation; avoiding action under either situation may be interpreted as missed care.

Studies have shown that patients demand comprehensive and understandable information and greater involvement in several aspects during the postoperative period [8, 29], This could reduce fear, stress, and anxiety [29]. Our study indicates high congruency of psychosocial care, while other studies have shown unmet psychosocial care needs in hospitals [10, 11]. Previous studies reported that patients lacked information about their care plans and the procedures they were going to undergo, and they wanted more discussion with nurses about their medication [8]. Further, patients wanted to be treated as individuals but often received standard treatment [30]. Lack of respect and dignity around privacy has been identified as an area of missed care; many patients feel that their confidentiality is not always respected during their conversations with nurses [31–36]. Further, dignity could be an issue in medical and surgical settings where men and women share the same bed bays and patients experience embarrassment when they are seen by nurses of the opposite sex [37, 38]. This could be less relevant in Norway and Denmark since the Nordic region scores highest regarding sexual equality worldwide, and equality between men and women is a fundamental value [39].

Our study revealed no association between educational level, sex; LOS, age, and how inpatients prefer to be respected, dignified, informed, and involved. Information, involvement, dignity, and respect are basic psychosocial needs, regardless of patient background. This aligns with Orique et al. [40], who found no significant association between patients’ education level and missed care; and contrasts Kalisch et al. [41], who found that patients with lower education levels reported more missed care than their counterparts.

Surveys that measure patient-reported experiences are one of several valid means for describing the quality of health services. It is important to consider patients’ perspectives throughout the healthcare process because patients are the principal end users of the services that health organizations provide [8]. The increasing international attention regarding patient-reported experiences as a quality indicator of patient care and safety reflects the ongoing commitment of the health service to involve patients and the public within the wider context in the development and evaluation of healthcare service delivery and quality improvement [42].

The Nordic countries are notorious for their strongly socialized healthcare, which include highly developed hospital services. A vital feature of the welfare state is that it aims at easy and equal access to adequate healthcare for the entire population. Healthcare systems in the Nordic countries are taxation-based and each citizen has equal access to the related services [43]. Further, patient-centered and person-oriented care has a long tradition in Scandinavia [44]; therefore, user participation and patients’ perspectives are among the cornerstones of the Nordic model and approach to healthcare provision [45], which might lead to more individualized care in Scandinavia. However, patients spend less time in hospitals than previously; and efficacy and productivity are essential in hospitals, with shorter posting time and faster hospitalization.

Employees are supposed to “do more for less.” Pressure on surgical care increases with the increasing number of operations performed per year, shorter hospital stays, and decreasing number of hospital beds [14–16]. Despite this, this study showed that patients experienced and perceived good psychosocial care. Further research related to FoC should include patients’ perspectives to inform nursing staff and ensure that nurses focus on truly important aspects and avoid potentially unnecessary work. It may also be interesting to differentiate between patient groups since patients with major complexity could benefit most from specific psychosocial treatment [4].

This study has limitations as 194 surgical inpatients is a rather small population, especially when they represent a wide range of ages and diagnoses. Additionally, answering a questionnaire on the day of discharge, when one may long to go home as quickly as possible, may limit the depth of the answers. Nevertheless, data collection on the day of discharge provides a fresh and immediate perspective on the patient’s experience, as the events are still recent in their memory. We do argue that this study is valuable since it brings new knowledge on surgical inpatients’ expectations and experiences with their psychosocial care needs through a rigorous method.

## Conclusion

This study of surgical inpatients’ experiences of psychosocial care assessed on the day of discharge revealed that patients in Norway and Denmark experience respectful and dignified treatment. They also felt involved and informed in their perioperative care. The average ratings regarding these aspects of psychosocial care needs were 72.1–93.8%. Including patient perspective in research promotes a patient-centered approach in practice. Patients can provide unique insights into their conditions, treatment experiences, and overall quality of care. From patients’ perspectives, we gain a deeper understanding of their needs, preferences, and priorities. This, in turn, enables the development of perioperative care that is more aligned with patient expectations, leading to improved patient-centered care.

Further, understanding patients’ preferences is a prerequisite for discussing improvements and quality of care with patients: this might clarify their expectations, thus leading to less stress and workload for nurses in hospitals. High ethical standards may lead nurses to provide unnecessary care to patients who may not even want it; this is indicative of discrepancies in nurses’ and patients’ priorities. Nurses should consider informing patients about why the care aspect is either important or unimportant in a given situation because overlooking either scenario may be interpreted as missed care.

## Data Availability

The data will be available from the corresponding author on request.

## List of Abbreviations

CI: Confidence interval
FoC: Fundamentals of care
HCAHPS: Hospital consumer assessment of healthcare providers and systems
LOS: Length of stay
PR: Perceived reality
QPP: Quality from the patient’s perspective
SDs: Standard deviations
SI: Subjective importance

## References

[1] Kitson A, Conroy T, Wengstrom Y, Profetto-McGrath J, Robertson‐Malt S (2010). Defining the fundamentals of care. Int J Nurs Pract.

[2] Mateo-Ortega D, Gómez-Batiste X, Maté J, Beas E, Ela S, Lasmarias C (2018). Effectiveness of psychosocial interventions in complex palliative care patients: a quasi-experimental, prospective, multicenter study. J Palliat Med.

[3] Caminiti C, Annunziata MA, Verusio C, Pinto C, Airoldi M, Aragona M (2021). Effectiveness of a psychosocial care quality improvement strategy to address quality of life in patients with cancer: the HuCare2 stepped-wedge cluster randomized trial. JAMA Netw Open.

[4] Szeverenyi C, Kekecs Z, Johnson A, Elkins G, Csernatony Z, Varga K (2018). The use of adjunct psychosocial interventions can decrease postoperative pain and improve the quality of clinical care in orthopedic surgery: a systematic review and meta-analysis of randomized controlled trials. J Pain.

[5] Carlson LE, Bultz BD (2003). Benefits of psychosocial oncology care: improved quality of life and medical cost offset. Health Qual Life Outcomes.

[6] Carlson LE, Bultz BD (2004). Efficacy and medical cost offset of psychosocial interventions in cancer care: making the case for economic analyses. Psychooncol.

[7] Kitson AL (2018). The fundamentals of care framework as a point-of-care nursing theory. Nurs Res.

[8] Bagnasco A, Dasso N, Rossi S, Galanti C, Varone G, Catania G (2020). Unmet nursing care needs on medical and surgical wards: a scoping review of patients’ perspectives. J Clin Nurs.

[9] Gustafsson N, Leino-Kilpi H, Prga I, Suhonen R, Stolt M (2020). Missed care from the patient’s perspective–a scoping review. Patient Pref Adhere.

[10] Albsoul R, FitzGerald G, Finucane J, Borkoles E (2019). Factors influencing missed nursing care in public hospitals in Australia: an exploratory mixed methods study. Int J Health Plann Manage.

[11] Ausserhofer D, Zander B, Busse R, Schubert M, De Geest S, Rafferty AM (2014). Prevalence, patterns and predictors of nursing care left undone in european hospitals: results from the multicountry cross-sectional RN4CAST study. BMJ Qual Saf.

[12] Mackie BR, Mitchell M, Marshall AP (2019). Patient and family members’ perceptions of family participation in care on acute care wards. Scand J Caring Sci.

[13] Rasmussen TS, Delmar C. Dignity as an empirical lifeworld construction—In the field of surgery in Denmark. Int J Qual Stud Health Well Being 2014;9(1):24849. 10.3402/qhw.v9.24849 10.3402/qhw.v9.24849 PMC4104009 25038001

[14] Broetje S, Jenny GJ, Bauer GF (2020). The key job demands and resources of nursing staff: an integrative review of reviews. Front Psychol.

[15] Moloney W, Boxall P, Parsons M, Cheung G (2018). Factors predicting registered nurses’ intentions to leave their organization and profession: a job demands-resources framework. J Adv Nurs.

[16] Thapa DR, Subedi M, Ekström-Bergström A, Josefsson KA, Krettek A (2022). Facilitators for and barriers to nurses’ work-related health-a qualitative study. BMC Nurs.

[17] van Belle E, Giesen J, Conroy T, van Mierlo M, Vermeulen H, Huisman-de Waal G (2020). Exploring person‐centred fundamental nursing care in hospital wards: a multi‐site ethnography. J Clin Nurs.

[18] Jangland E, Teodorsson T, Molander K, Muntlin Athlin Ã (2018). Inadequate environment, resources and values lead to missed nursing care: a focused ethnographic study on the surgical ward using the Fundamentals of Care framework. J Clin Nurs.

[19] Van Humbeeck L, Malfait S, Holvoet E, Vogelaers D, De Pauw M, Van Den Noortgate N (2020). Value discrepancies between nurses and patients: a survey study. Nurs Ethics.

[20] Schopp A, Leino-Kilpi H, Välimäki M, Dassen T, Gasull M, Lemonidou C (2003). Perceptions of privacy in the care of elderly people in five european countries. Nurs Ethics.

[21] McCormack B, McCance T. Person-centred nursing. Theor Pract. Oxford: Wiley-Blackwell. 2010.

[22] Kymre IG, Uhrenfeldt LU, Pedersen MK, Ingstad K, Pedersen PU (2023). Development and validation of the perioperative care and user participation (POUP) questionnaire. Scand J Caring Sci.

[23] Feo R, Conroy T, Alderman J, Kitson A (2017). Implementing fundamental care in clinical practice. Nurs Stand.

[24] Wilde Larsson BW, Larsson G (2002). Development of a short form of the quality from the patient’s perspective (QPP) questionnaire. J Clin Nurs.

[25] FHI. Pasienters erfaringer med norske sykehus i 2019. Metodebeskrivelse og analyser for landet samlet. Norway: Oslo, 839. 2020; 2019. Folkehelseinstituttet Rapport. https://www.fhi.no/contentassets/1ed1cf501b0a43d58415dcbb3ab889e6/metodebeskrivelse-og-analyser-for-landet-samlet.pdf

[26] Danske Regioner. LUP-somatik. Seks ud af syv patienter: ”Vi er meget tilfredse”; 2022. https://www.regioner.dk/services/nyheder/2022/marts/seks-ud-af-syv-patienter-vi-er-meget-tilfredse

[27] Blomberg AC, Bisholt B, Lindwall L (2018). Responsibility for patient care in perioperative practice. Nurs Open.

[28] Blomberg AC, Lindwall L, Bisholt B (2019). Operating theatre nurses’ self-reported clinical competence in perioperative nursing: a mixed method study. Nurs Open.

[29] Gobbo M, Saldaña R, Rodríguez M, Jiménez J, García-Vega MI, de Pedro JM (2020). Patients’ experience and needs during perioperative care: a focus group study. Patient Prefer Adherence.

[30] Kaptain K, Ulsøe ML, Dreyer P (2019). Surgical perioperative pathways-patient experiences of unmet needs show that a person-centred approach is needed. J Clin Nurs.

[31] Abdel Maqsood AS, Oweis AI, Hasna FS (2012). Differences between patients’ expectations and satisfaction with nursing care in a private hospital in Jordan. Int J Nurs Pract.

[32] Alasad J, Tabar NA, AbuRuz ME (2015). Patient satisfaction with nursing care: measuring Outcomes in an international setting. J Nurs Admin.

[33] Crispin V, Bugge C, Stoddart K (2017). Sufficiency and relevance of information for inpatients in general ward settings: a qualitative exploration of information exchange between patients and nurses. Int J Nurs Stud.

[34] Lemonidou C, Merkouris A, Leino-Kilpi H, Välimäki M, Dassen T, Gasull M (2003). A comparison of surgical patients’ and nurses’ perceptions of patients’ autonomy, privacy and informed consent in nursing interventions. Clin Eff Nurs.

[35] Ringdal M, Chaboyer W, Ulin K, Bucknall T, Oxelmark L (2017). Patient preferences for participation in patient care and safety activities in hospitals. BMC Nurs.

[36] Valizadeh F, Ghasemi SF (2020). Human privacy respect from viewpoint of hospitalized patients. Eur J Transl Myol.

[37] Ebrahimi H, Torabizadeh C, Mohammadi E, Valizadeh S (2012). Patients’ perception of dignity in iranian healthcare settings: a qualitative content analysis. J Med Ethics.

[38] Mbuzi V, Fulbrook P, Jessup M (2017). Indigenous cardiac patients’ and relatives’ experiences of hospitalisation: a narrative inquiry. J Clin Nurs.

[39] World Economic Forum. Global gender gap report 2021. Insight report March 2021. file:///C:/Users/01700176/OneDrive%20-%20Nord%20universitet/FORSKNING/CocKTaL%20Forskningsgruppe%20Lisbeth%20U/KUPP%20Sp%C3%B8rreunders%C3%B8kelse/Artikkel%202%20FoC%20sosiokulturell/Relevante%20artikler/Closing%20gender%20gap.pdf. Switzerland: Geneva.

[40] Orique SB, Patty CM, Sandidge A, Camarena E, Newsom R (2017). Quantifying missed nursing care using the hospital consumer assessment of healthcare providers and systems (HCAHPS) survey. J Nurs Adm.

[41] Kalisch BJ, Xie B, Dabney BW (2014). Patient-reported missed nursing care correlated with adverse events. Am J Med Qual.

[42] Weldring T, Smith SM (2013). Patient-reported outcomes (pros) and patient-reported outcome measures (PROMs). Health Serv Insights.

[43] Hafsteinsdóttir TB (2019). Introduction on leadership, nursing and the nordic countries. Leadership in nursing: experiences from the european nordic countries.

[44] Uhrenfeldt L, Sørensen EE, Bahnsen IB, Pedersen PU (2018). The centrality of the nurse-patient relationship: a scandinavian perspective. J Clin Nurs.

[45] Storm M, Coulter A, Schibevaag KA (2017). Patient-centred care in the nordic countries. Researching patient safety and quality in healthcare: a nordic perspective.

[46] Helse- Og Omsorgsdepartementet. Lov om helsepersonell mv (helsepersonelloven), 64. ; 1999. https://lovdata.no/dokument/NL/lov/1999-07-02

